# The caries preventive effect of 1-year use of low-dose xylitol chewing gum. A randomized placebo-controlled clinical trial in high-caries-risk adults

**DOI:** 10.1007/s00784-017-2075-5

**Published:** 2017-03-16

**Authors:** Fabio Cocco, Giovanna Carta, Maria Grazia Cagetti, Laura Strohmenger, Peter Lingström, Guglielmo Campus

**Affiliations:** 1 Department of Surgery, Microsurgery and Medical Sciences—School of Dentistry, University of Sassari, Viale San Pietro 43/C, I-07100 Sassari, Italy; 2 WHO Collaborating Centre of Milan for Epidemiology and Community Dentistry, University of Milan, Milan, Italy; 3 Department of Biomedical, Surgical and Dental Sciences, University of Milan, Milan, Italy; 4 Department of Cariology, Institute of Odontology, The Sahlgrenska Academy, University of Gothenburg, Gothenburg, Sweden

**Keywords:** Dental caries, Preventive medicine, Xylitol, Chewing gums, RCT

## Abstract

**Objectives:**

The caries preventive effect of long-term use (1 year) of low-dosage (2.5 g/die) of xylitol chewing gum in a high-caries-risk adult population was evaluated.

**Materials and methods:**

In this randomized clinical trial, 179 high-caries-risk adults were assigned to two experimental groups, xylitol and polyols. Caries status, salivary mutans streptococci (MS), and plaque pH were re-evaluated after 2 years from baseline in 66 xylitol and 64 polyol subjects. Outcomes (the net caries increment for initial, moderate, and extensive caries lesions and for the caries experience) were evaluated using the nonparametric Mann–Whitney *U* test.

**Results:**

The total caries experience increment was 1.25 ± 1.26 in the xylitol group and 1.80 ± 2.33 in the polyol group (*p* = 0.01). Subjects treated with xylitol chewing gums had a reduction of risk rate at tooth level of 23% with respect to those treated with polyols with a number needed to treat of 55 teeth. The area under the curve at pH 5.7 was statistically significantly lower (*p* = 0.02) during the experimental period in the xylitol group. A decrease of the concentration of salivary MS was noted in the xylitol group (*p* < 0.01).

**Conclusions:**

Subjects using the low-dose xylitol chewing gum showed a significantly lower increment of initial and extensive caries lesions and overall a lower increment of caries experience.

**Clinical relevance:**

One-year use of chewing gums provides an effective means for the prevention of caries disease.

**Trial registration number:**

NCT02310308

## Introduction

Modern concepts regard caries as an interaction between host and environmental factors, where biological, social, and behavioural factors are expressed in a highly complex interactive manner with the dental biofilm as the key element [1]. Dietary fermentable carbohydrates are the main triggering factor for development of cariogenic biofilm [2]. When biofilm is matured, the presence of sugars promotes a higher plaque cariogenicity, keeping frequently pH value under the critical levels for the demineralization of enamel and dentine [3, 4]. The acidic environment within biofilm favours the growth of more acid-tolerant bacteria such as mutans streptococci (MS) and lactobacilli [5]. Preventive strategies are needed and recommended to control caries risk factors, mainly based on dietary changes, i.e. sweetener intake reduction, and enhancing host resistance, i.e. twice-daily use of fluoride toothpastes [6, 7].

However, the high-skewed caries prevalence distribution suggests the need of developing new and effective preventive approaches especially for high-risk groups [8, 9]. The use of sugar-free chewing gums may contribute to prevent dental caries [6, 10]. The increase of stimulated saliva flow rate promotes oral clearance and enhances the buffering capacity to neutralize plaque pH [11]. It is described in literature that the consumption of xylitol can lead to less plaque, less numbers of MS, and a lower caries increment [6, 12, 13]. Although the xylitol mechanisms are not fully known, several studies demonstrate its benefits and clinical trials have shown that xylitol possesses both noncariogenic and cariostatic properties [6, 10, 12, 14]. There is a debate on the effective dose; a total daily dose from 3 to 8 g of xylitol is usually recommended for a clinical effect. In children, the efficacy of low doses of xylitol in caries prevention was speculated [15]; anyhow, the results are inconclusive for long-term effectiveness regarding MS and caries reduction [16].

The existing evidence of xylitol role in caries prevention needs to be supplemented by well-designed randomized controlled trials, especially in adults at high risk of caries.

Starting from this premise with the aim to assess the caries preventive effect of long-term use of low dosage of xylitol chewing gums in a high-caries-risk adult population, a randomized placebo-controlled clinical trial with two arms was designed and carried out.

## Methods

This paper reports on findings obtained in a larger research project that examines the effect of several functional foods supplied through chewing gums on caries prevention in an adult population. This paper was focused on the effect of low-dose xylitol.

### Ethical approval

The present study was carried out in Sassari (Italy) under the supervision of the WHO Collaborating Centre for Epidemiology and Community Dentistry of Milan, Italy, and lasted from September 2012 to June 2015. The study was designed as a randomized clinical trial, approved by the Ethics Committee of the University of Sassari (n°1083/L 23/07/2012), and registered (Protocol Registration Receipt NCT02310308) at http://www.clinicaltrial.gov.

### Study population

Data from the Italian National Institute for Statistics for 2011 gave the number of 30–45-year olds living in the town of Sassari as 22,614.

The inclusion criteria were as follows: age range between 30 and 45 years; presence of a minimum of 12 natural teeth; presence of at least one cavitated (D_2/3_) caries lesion, but not more than three; a salivary concentration of MS equal or upper to 10^5^ CFU/mL saliva; no current periodontitis (no sites of probing pocket depth ≥5 mm or attachment loss of ≥2 mm, apart from gingival recession); absence of dysfunction of temporomandibular joint; good health systemically as assessed by a medical questionnaire; no allergy to any of the ingredients of the study products; no orthodontic banding or removable prosthesis; and no use of antibiotics or participation in a clinical study in the previous 30 days. The use of antibiotics was also recorded at interim examinations (6, 12, and 24 months) before saliva sampling, and subjects using antibiotics 30 days before the evaluation were excluded.

Sample size for preliminary screening was performed through G*Power 3.1.3 for Apple using logistic regression with an odds ratio of 1.8 and an error probability of 0.04; the total sample was set at 312.

In order to get statistical comparable results, the number of subjects per group to be included in the analysis was calculated. Considering a 40% difference among groups to be significant and a 95% probability of obtaining a significant difference between groups at the 5% severity, the resulting number of subjects per group was set in 64.

**Fig. 1:**
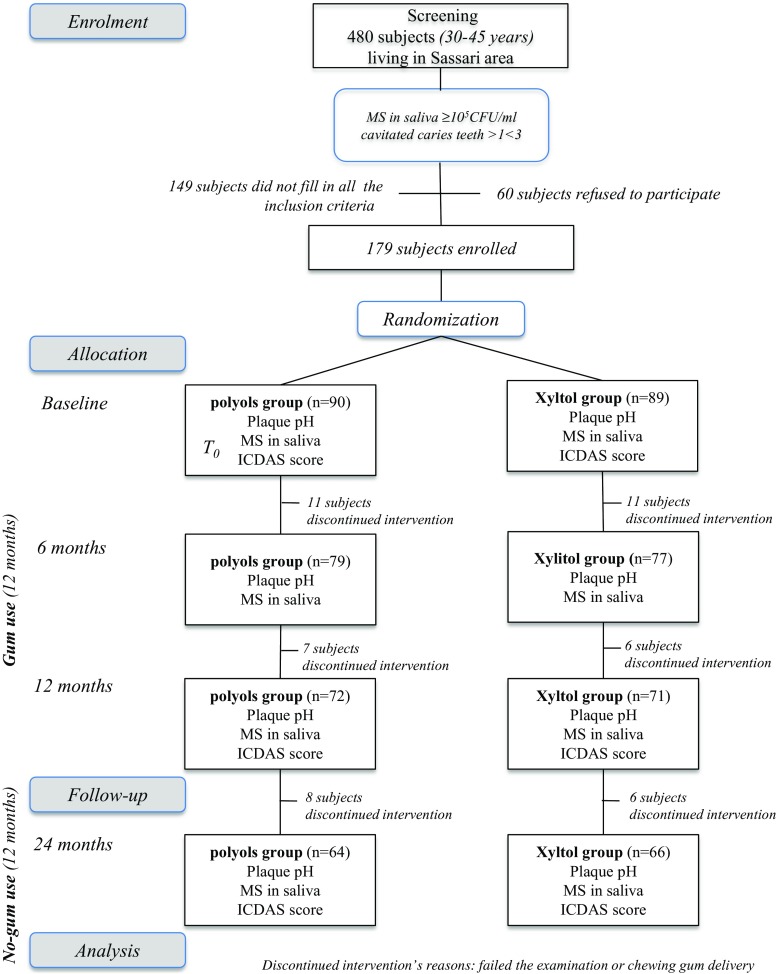
Flow chart of the study

With the collaboration of the municipal electoral registry office, a letter explaining the purpose of the study and the informed consent were randomly distributed to 5% (1131 subjects) of the age group considered living in Sassari. A total of 480 subjects (42.4% acceptance rate) accepted to participate and were examined for conditions that would preclude participation. The flow chart, displayed in Fig. 1, shows the design of the study.

Randomization was performed (GCampus) using Excel 2014 in permuted blocks of two or four with random variation of the blocking number, and two groups were created: (1) the first group received gums containing the same mixture of polyols except xylitol; 2) the second group received gums containing the same polyols mixture plus a low amount of Xylitol. The clinical examination was repeated at the end of the chewing gum administration period (12 months) and at the end of the experimental period (24 months).

A benchmark examiner (GCampus) trained and calibrated one examiner (GCarta) that performed all dental screenings. Baseline training consisted in 1-day (6 h) theoretical course, followed by examination of 54 extracted teeth plus a session of 120 photographs of extracted teeth. Two days after the theoretical course, a clinical training involving examination of 55 adults was performed. The subjects were re-examined after 72 h. Inter-examiner reliability with the “benchmark” (GCampus) was evaluated using fixed-effect analysis of variance. Intra-examiner reproducibility was assessed as the percentage of agreement using Cohen’s kappa statistic [17]. Good reliability was found between examiner and benchmark (*p* = 0.15) with a low mean square of error (0.47). Intra-examiner reliability was also high (Cohen’s kappa = 0.88). Interim and follow-up training was also performed. At the interim clinical evaluation (12 months), 47 adults not enrolled into the trial were re-examined after 72 h with a good reliability between examiner and benchmark (*p* = 0.14) with a mean square of error (0.49) and a high intra-examiner reliability (Cohen’s kappa = 0.91). Before the final follow-up examination (24 months), 45 adults not enrolled into the trial were re-examined after 72 h with an inter-examiner reliability (examiner vs benchmark (*p* = 0.14) with a mean square of error (0.49) and a high intra-examiner reliability (Cohen’s kappa = 0.91). Subjects were examined using a mouth mirror and a Community Periodontal Index probe (approved by the WHO) under optimal lighting. The ICDAS (International Caries Detection and Assessment System) index was used to register caries at tooth level as initial or moderate or extensive lesions and the number of filled and missing teeth for caries [18]. Initial caries lesion can be defined as a primary lesion, which has not reached the stage of an established lesion with cavitation. Moderate caries lesions are defined as white or brown spot lesion with localized enamel breakdown or an underlying dentine shadow without visible dentine exposure. Severe caries lesions are defined as distinct cavity in opaque or discoloured enamel with visible dentine [19]. Those participants who referred to consume more than three pieces of sugar-free chewing gum a day were excluded. The elected participants agreed not to consume any other chewing gums than those supplied for the study.

All participants were residents in an area with a low natural fluoride content in the drinking water (0.04 mg/L) (http://www.abbanoa.it/distretto-6-sassari1), but they reported to use a fluoridated toothpaste on a regular basis.

### Microbiological evaluation

Immediately after the clinical assessment, an evaluation of MS concentration in saliva was performed. Nonstimulated whole saliva was collected over 150 s in sterile vials (Nunc, Kamstrup, Denmark). The samples were transported to the Department of Microbiology and processed within 45 min after collection. The samples were serially diluted in sterile PBS (Sigma Chemicals, St. Louis, MO, USA). Aliquots of 5 μL were inoculated on mitis salivarius bacitracin agar, a medium that at concentrations of bacteria 1 × 10^3^ to 1 × 10^10^/mL shows a good sensitivity and selectivity in MS detection. The plates were incubated in a 5% CO_2_ environment at 37 °C for 72 h after which the colony-forming units were identified by morphology, size, and colour and counted in a stereomicroscope.

### Plaque pH measurements

Inter-proximal plaque pH of each subject was evaluated using pH indicator strips [20], which measure a pH value in the range 4.0–7.0 (Spezialindikator, pH range 4.0–7.0; Merck, Darmstadt, Germany), with a resolution of 0.2–0.5 pH unit; in addition, the strips are easy to use. The strips were cut into four pieces (approx. 2 mm in width) in order to be more easily inserted into the inter-proximal space and held into the inter-dental space for 10 s, after which they were removed and their colour compared to the colour index scheme supplied by the manufacturer. The pH was determined to one decimal of the value.

Plaque pH was assessed at baseline, after 6 and 12 months of chewing gum use and 12 months after the cessation of chewing gum use. For each subject, three measurements were carried out on two sites, between the second premolar and the first molar, right and left of the upper jaw; the average pH value was later calculated. Recordings were performed before and at 2, 5, 10, 15, 20, and 30 min after a mouth rinse with 10% sucrose and carried out by one examiner (FC). Area under the curve (AUC), described as the area between reference pH (6.2 or 5.7) line and the pH curve, was calculated using a computer-based program [21]. The area under the curve at pH 5.7 and 6.2 (AUC_5.7_ and AUC_6.2_) was used as a reference for the dissolution of enamel and dentine, respectively.

### Treatment and sample collection

Overall, 480 subjects were examined, 331 fulfilled the inclusion criteria, and 179 accepted to be enrolled in the trial.

All chewing gums were produced and supplied by Perfetti Van Melle SpA (Lainate, Italy). The polyol chewing gum was sugar-free containing 28% isomalt, 31% sorbitol, 9% mannitol, and 1% maltitol syrup. Xylitol chewing-gum contained 30% of Xylitol, 26% Sorbitol, 11% Mannitol and 1% Maltitol syrup.

All chewing gums weighed 1.4 g each and were identical in colour, shape, and taste. Chewing gums were supplied in plain white containers coded as ‘green’ or ‘blue’ according to the group. The code was sealed by an independent monitor and not broken until the statistical analysis was finalized.

The subjects were instructed to chew for 5 min two pellets in the morning, two after the midday meal, and one in the afternoon; the total daily intake of xylitol was 2.5 g/day. The subjects were asked to make no changes in their dietary and oral hygiene habits. Tooth brushing was not allowed for at least 1 h after the use of chewing gums. All subjects received a fluoridated toothpaste containing 1450 ppm NaF (Mentadent P; Unilever Italia, Milan, Italy) to be used during the experimental period. They were also asked to avoid any other oral hygiene adjuvant and any commercial xylitol or sorbitol product throughout the study period. The body’s tolerance to different polyols was assessed by means of a questionnaire administered to the participants shortly after the gum distribution had started and 6 months later, while the study was still proceeding. The questions focused on the potential side effects of using the gum. In order to evaluate the success of the chewing gum intake, participants were given chewing gums necessary for a single month at a time and asked to return the empty packs when receiving those for the following month.

### Statistical analysis

The tooth as the unit of analysis was evaluated as follows: first, the net caries increment for initial (ICDAS 1 and 2), moderate (ICDAS 3 and 4), and extensive (ICDAS 5 and 6) caries severity using ICDAS (Δ-initial, Δ-moderate, and Δ-extensive) was calculated. The number of events is the sum of the Δ-caries change of status recorded at baseline, 12 months of gum use, and after 12 months of no-gum use. Secondly, caries experience expressed as the sum of extensive caries lesions plus the number of filled and extracted teeth due to caries was calculated and consequently the caries increment was recorded. Differences across mean number of events between groups for each variable were evaluated using the nonparametric Mann–Whitney *U* test.

The data on inter-proximal plaque pH at baseline, 6 months of gum use, 12 months of gum use, and after 12 months of no-gum use were analysed for statistically significant differences using repeated measures of ANOVA.

Differences in proportion relating to microbiological counts at baseline and follow-ups were assessed using equality of proportion test. The lowness curve was used to describe the trend of plaque pH and salivary MS. The effectiveness of the treatment was assessed for those who fully followed the protocol (per-protocol subjects) by calculating the reduction in risk ratio (RRR) and the related number needed to treat (NNT) value [22]. An event was defined as the change of status at tooth level, i.e. the development of a new lesion or the progression of an existing lesion to a more severe stage. All data were analysed using the software STATA® (v13 for Mac). For all statistical analyses, the statistical significance was set at *α* = 0.05.

## Results

A total of 130 subjects (72.62% of the initial sample) completed the trial (66 in the xylitol group and 64 in the polyol group). In Fig. 1 is reported the flow chart of the study with the number of the dropout subjects; the highest number of dropouts was reported at the 6-month evaluation. The main reason for dropping out was the discontinued intervention. No side effects were observed in any subjects. Moreover, the use of chewing gums after the experimental period was fairly insignificant; only 19 (10.61%) subjects reported the regular use (once a day or more) of sugar-free chewing gum (*data not in table*). The (Δ) caries values as the mean number of events at the different time points of the trial (gum use 0–12 months, no-gum use 12–24 months, and between baseline and 24 months) are reported in Table 1. The net caries increment for initial, moderate, and extensive caries lesions using ICDAS (Δ-initial, Δ-moderate, and Δ-extensive) is displayed. No statistically significant differences between groups were recorded at baseline, and no gender difference for each Δ-value was also observed (*data not in table*). During the gum use, the Δ-values were statistically significantly different between groups for initial caries lesions (polyol 0.09 ± 0.15 and xylitol 0.05 ± 0.12, *p* = 0.01) and for extensive caries lesions (polyol 0.18 ± 0.38 and xylitol 0.11 ± 0.12, *p* = 0.02), while no statistical differences (*p* = 0.18) were recorded for moderate caries lesions between groups. In the no-gum use period, no statistically significant differences were recorded between the two groups for each caries severity level. Regarding the total duration of the trial (0–24 months), the Δ-values were statistically significantly different for initial caries lesions (polyol 0.20 ± 0.67 and xylitol 0.14 ± 0.37, *p* = 0.01) and for extensive caries lesions (polyol 0.44 ± 0.73 and xylitol 0.30 ± 0.52, *p* = 0.03). No statistically significant difference in the comparison between the two groups regarding moderate caries lesions was observed (*p* = 0.12). The total caries experience increment was 1.80 ± 2.33 in the polyol group and 1.25 ± 1.26 in the xylitol group (*p* = 0.01).

**Table 1:**
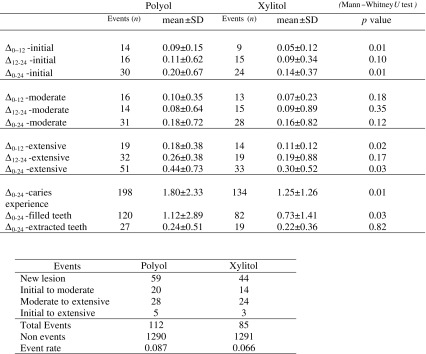
The (Δ) caries values as the mean number of events measured between baseline and 12-month follow-up examinations (Δ_0–12_), between 12-month follow-up and 24-month follow-up (Δ_12–24_), and between baseline and 24-month follow-up (Δ_0–24_)

The difference was evaluated for caries severity (initial, moderate, and extensive) and caries experience (extensive caries plus the number of filled and extracted teeth due to caries). The effectiveness of the treatment was assessed for those who fully followed the protocol (per-protocol subjects) by calculating the reduction in risk ratio (RRR) and the related number needed to treat (NNT)-value. An event was defined as the change of status at tooth level

*χ* ^2^ *=* 3.18; *p =* 0.07; RRR *=* 0.23; _95%_CI = −0.02/0.41; NNT = 55; _95%_CI = −974/27

Subjects treated with xylitol chewing gums had a reduction of risk (RRR) rate at tooth level of 23% with respect to those treated with polyols, with a NNT of 55 teeth. The event rate was 0.087 in the polyol group and 0.066 in the xylitol group. The chi-squared test was 3.18 (*p* = 0.07).

At baseline, plaque pH and salivary MS concentration were similar in the groups, with no statistically significant differences. Table 2 displays the mean ± standard deviation for AUC_5.7_ and AUC_6.2_ during the trial. At baseline, the curves of AUC_5.7_ and AUC_6.2_ were similar in the groups. The AUC_5.7_ was statistically significantly different (*p* = 0.02) during the experimental period in the xylitol group, ranging from 11.5 ± 0.5 at baseline to 9.8 ± 0.3 at the 2-year evaluation. The comparison between groups was statistically significantly different (*p* = 0.04) only at the end of the experimental period. The AUC_6.2_ was statistically significantly different (*p* < 0.01) in the xylitol group, ranging from 22.8 ± 0.6 at baseline to 18.7 ± 0.6 at the 2-year evaluation_._ No statistically significant differences were observed between groups. The minimum pH showed small variations among the four time points for the xylitol group, whereas in the polyol group, a decrease was detected at 1- and 2-year evaluations. Statistically significant differences were observed in the xylitol group for both curves (*β* = 0.031, _95%_CI = 0.003/0.081, and *p* = 0.01 for the AUC_5.7_ and *β* = 1.167, _95%_CI = −0.182/2.538, and *p* = 0.09 for the AUC_6.2_).

**Table 2:** Plaque pH: AUC_5.7_ and AUC_6.2_ (mean ± SD) during the experimental period

AUC_5.7_						
	Time	Polyol		Xylitol		*p* value one-way ANOVA
		Subjects (*n*)	Mean ± SD	Subjects (*n*)	Mean ± SD	
	Baseline	90	11.7 ± 0.6	89	11.5 ± 0.5	0.87
Gum use	6 months	79	11.4 ± 0.5	77	11.3 ± 0.3	0.90
	12 months	72	9.9 ± 0.3	71	9.6 ± 0.4	0.78
No-gum use	24 months	64	11.4 ± 0.2	66	9.8 ± 0.3	0.04
	*p* value one-way ANOVA		0.08		0.02	
AUC_6.2_						
	Time	Polyol		Xylitol		*p* value one-way ANOVA
		Subjects (*n*)	Mean ± SD	Subjects (*n*)	Mean ± SD	
	Baseline	90	21.6 ± 0.2	89	22.8 ± 0.6	0.11
Gum use	6 months	79	18.9 ± 0.8	77	20.1 ± 0.5	0.07
	12 months	72	19.0 ± 0.8	71	18.6 ± 0.7	0.26
No-gum use	24 months	64	19.5 ± 0.2	66	18.7 ± 0.6	0.15
	*p* value one-way ANOVA		0.12		<0.01	

Salivary bacteria (MS) results are shown in Table 3. At baseline, all the subjects had a MS concentration ≥10^5^ CFU/mL; this was also one of the inclusion criteria for enrolment in the trial. In both groups, the bacterial concentration decreases, but only in the xylitol group the decrease was statistically significant (*p* < 0.01); anyhow, comparison between groups was statistically significant both at the end of gum use period and at the end of the no-gum use period (*p* = 0.03 and *p* = 0.04, respectively). A statistically significant trend in the MS reduction was observed in the xylitol group (*β* = 0.031, _95%_CI = 0.003–0.081, and *p* = 0.01).

**Table 3:** Concentration of salivary MS (log_10_ CFU/mL in saliva: mean ± SE) at the different time points (baseline, 6 months of gum use, 12 months of gum use, and after 12 months of no-gum use) in the two groups

	Time	Polyol		Xylitol		*p* value one-way ANOVA
		Subjects (*n*)	Mean ± SD	Subjects (*n*)	Mean ± SD	
Gum use	Baseline	90	5.32 ± 0.43	89	5.41 ± 0.35	0.29
	6 months	79	5.22 ± 0.21	77	5.33 ± 0.46	0.31
	12 months	72	5.33 ± 0.42	71	5.16 ± 0.42	0.03
No-gum use	24 months	64	5.33 ± 0.46	66	5.15 ± 0.64	0.04
	*p* value one-way ANOVA		0.42		<0.01	

## Discussion

The purpose of this study was to assess the caries preventive effect of long-term use of low-dosage xylitol administered through a chewing gum in a caries high-risk adult population. To elucidate this aim, a two-arm randomized controlled intervention trial was designed and carried out.

The use of sugar substitutes is an intervention able to reduce caries risk [23, 24], and several nonfermentable sweeteners are incorporated into many products, such as chewing gums, lozenges, candies, and syrup, with the xylitol as the main substitute [15]. Several studies indicated an effect, dose- and frequency-dependent, of xylitol with a total daily dose from 3 to 8 g a day and a frequency of four to five times a day [16, 25].

In this trial, two types of chewing gums were studied: a low dosage (0.5 g/pellet) of xylitol chewing gum and a polyol (isomalt, sorbitol, mannitol, and maltitol syrup) chewing gum. The trial focused on caries increment (Δ-initial, Δ-moderate, and Δ-extensive caries lesions and Δ-caries experience), plaque acidogenicity, and mutans streptococci concentration in saliva. Two clinical caries evaluations were performed, the first at the end of the chewing gum use period (12 months from baseline) and the second at the end of the no-gum use period (24 months from baseline), during which no other community-based caries prevention strategies were pursued in the sample, except for the personal oral hygiene habits.

At the end of the gum use period, statistically significant differences regarding initial and extensive Δ-caries values were observed between the two groups. Subjects using the low-dosage xylitol chewing gum showed significantly lower caries increment. The comparison between the two follow-up evaluations (12 and 24 months from baseline) showed no statistically significant differences between the two groups, but the comparison with respect to baseline was still statistically significant. Subjects using the low-dosage xylitol chewing gum showed a significantly lower increment of initial and extensive caries lesions and overall a lower increment of caries experience. Few studies reported a long-term caries preventive effect associated with xylitol gum that persists or even increases over time [12, 26]. In this trial, the long-term effect might be ascribed to the high statistically significant values observed at the end of the chewing gum period (12 months) and these statistically significant values persist even at the 24-month evaluation.

Different results were reported in a recent multicenter placebo-controlled randomized trial [27], where adults consumed 5 g daily of xylitol or placebo lozenges during a 33-month period. No significant effect was observed in the xylitol group regarding reduction in caries increment even if a dose almost double than those administered in the present study was used. Several hypotheses might be speculated to assess these different results. Although the two studies were similar regarding the number of subjects, the sample of the present study was highly homogeneous (age range, caries risk factors, and number of caries). Moreover, the total caries figure was included in the present study, considering even initial lesions and missed teeth due to caries, giving a wider picture. In addition, timing and administration modalities were different in the two trials; in the present one, xylitol was administered via a chewing gum; this led to the release of xylitol times higher than that of other studies. Xylitol was administered via a chewing gum far from main meals, allowing the polyol to act for a quite long period, and and due to a prolonged residence time of xylitol in the oral cavity.

Plaque pH comparison between groups was statistically significantly different, showing an area under the curve for enamel dissolution less pronounced in the xylitol group at the follow-up evaluations. Regarding the area under the curve for dentine dissolution, no statistically significant difference between groups was recorded, but a decrease in the area was noted during the entire experimental period only in the xylitol group. This pH figure reflects the trend of cariogenic bacteria during the trial period. A statistically significant difference in mutans streptococci concentration was evident between groups both at the end of the chewing gum use period and at the end of the no-gum use period. A growth-reducing effect of xylitol on salivary MS has been described, suggesting a long-term efficacy [24]. This finding seems to demonstrate a prolonged effect of Xylitol on different plaque-related variables, acidogenicity, and bacteria concentration, even if the clinical efficacy seems to be limited at the gum use period.

Some limits of the study design need to be underlined. First of all, the study population belonged to an age range, in which the habit to chew daily chewing gum is not common, so it was difficult to find a complete compliance and this might have affected the dropout. The number of subjects and the inclusion criteria do not allow to generalize the results to the general population of this age group. It is disputable if the results of this trial may not overlap to younger populations, taking into consideration that the majority of studies were carried out on children. A quite important issue is to determine if the preventive effect is mainly due to xylitol amount or the frequency of use. The results of the present study put some tiles to the importance of the frequency of xylitol use; anyway, more trials are needed to elucidate this aspect.

Although several studies were carried out on the caries preventive effect of Xylitol, this study holds almost unique characteristics like the age of the subjects (adult population), the length of the administration and the follow-up (12 and 24 months, respectively), the low dosage of xylitol administered (2.5 g/die), and the administration vehicle (chewing gums).

In conclusion, a long use of xylitol chewing gums with low concentration of polyol, controlling cariogenic bacteria concentration and plaque acidogenicity, provides an effective means for the prevention of caries disease.
